# The effects of exceeding low-risk drinking thresholds on self-rated health and all-cause mortality in older adults: the Tromsø study 1994–2020

**DOI:** 10.1186/s13690-023-01035-0

**Published:** 2023-02-16

**Authors:** Line Tegner Stelander, Geir Fagerjord Lorem, Anne Høye, Jørgen G. Bramness, Rolf Wynn, Ole Kristian Grønli

**Affiliations:** 1 Division of Mental Health and Substance Abuse, University Hospital of North Norway, P.O. Box 6124, 9291 Tromsø, Norway; 2 Department of Clinical Medicine, Faculty of Health Sciences, UiT The Arctic University of Norway, Tromsø, Norway; 3 Department of Psychology, Faculty of Health Sciences, UiT The Arctic University of Norway, Tromsø, Norway; 4 Norwegian Institute of Public Health, Oslo, Norway; 5 Norwegian National Advisory Unit on Concurrent Substance Abuse and Mental Health Disorders, Hamar, Norway

**Keywords:** Alcohol consumption, Older adults, Self-rated health, Mortality, Mental distress, Longitudinal study, The Tromsø study, Norway

## Abstract

**Background:**

Based on findings of increasing alcohol consumption in older adults, it is important to clarify the health consequences. Using data from the Tromsø study, we aimed to investigate the relationship between different levels of alcohol consumption in old adulthood and self-rated health trajectories and all-cause mortality.

**Methods:**

This is an epidemiological study utilizing repeated measures from the Tromsø study cohort. It allows follow-up of participants from 1994 to 2020. A total of 24,590 observations of alcohol consumption were made in older adults aged 60–99 (53% women). Primary outcome measures: Self-rated health (SRH) and all-cause mortality. SRH was reported when attending the Tromsø study. Time of death was retrieved from the Norwegian Cause of Death Registry. The follow-up time extended from the age of study entry to the age of death or end of follow-up on November 25, 2020. Predictor: Average weekly alcohol consumption (non-drinker, < 100 g/week, ≥100 g/week). We fitted two-level logistic random effects models to examine how alcohol consumption was related to SRH, and Cox proportional hazards models to examine its relation to all-cause mortality. Both models were stratified by sex and adjusted for sociodemographic factors, pathology, biometrics, smoking and physical activity. In addition, all the confounders were examined for whether they moderate the relationship between alcohol and the health-related outcomes through interaction analyses.

**Results:**

We found that women who consumed ≥100 g/week had better SRH than those who consumed < 100 g/week; OR 1.85 (1.46–2.34). This pattern was not found in men OR 1.18 (0.99–1.42). We identified an equal mortality risk in both women and men who exceeded 100 g/week compared with those who consumed less than 100 g/week; HR 0.95 (0.73–1.22) and HR 0.89 (0.77–1.03), respectively.

**Conclusions:**

There was no clear evidence of an independent negative effect on either self-rated health trajectories or all-cause mortality for exceeding an average of 100 g/week compared to lower drinking levels in this study with up to 25 years follow-up. However, some sex-specific risk factors in combination with the highest level of alcohol consumption led to adverse effects on self-rated health. In men it was the use of sleeping pills or tranquilisers and ≥ 20 years of smoking, in women it was physical illness and older age.

**Supplementary Information:**

The online version contains supplementary material available at 10.1186/s13690-023-01035-0.

## Background

Older adults in most western European countries have increased their alcohol consumption, but little is known about how increased drinking in old adulthood affect all-cause mortality and health-related quality of life [1–6]. Excessive alcohol consumption is associated with acute harms such as falls, injuries, and confusion as well as long-term effects linked to many diseases common in older adults [5, 7, 8]. Although it has been widely accepted that a J- or U-shaped association exists between alcohol consumption, cardiovascular disease, type 2 diabetes and all-cause mortality, with a lower risk for moderate drinkers compared to abstainers or heavy drinkers [9–13], recent evidence casts doubt on whether any beneficial health effect of alcohol exists [14–18]. Albeit the lack of international consensus on low-risk drinking guidelines, there is a need to study possible at-risk drinking thresholds in older adults separately. Lean body mass and total body water decrease with increasing age, resulting in higher levels of blood alcohol from the same amount of alcohol compared to younger people [5]. Additionally, due to higher prevalence of prescription and over-the-counter medications and increasing somatic and mental illnesses with increasing age, research on the health consequences of alcohol consumption in older adults is necessary [19–21]. However, some studies indicate that older adults may tolerate alcohol just as well as their younger counterparts [22–24]. Nevertheless, a life situation altered by retirement; illness; loss of a spouse, partner, family members or friends; loneliness; or hopelessness may facilitate negative health consequences of alcohol consumption [5, 25]. Despite sparse evidence that older adults may tolerate alcohol quite well, an increase has been reported in alcohol-related hospital admissions among older adults [6, 26, 27]. Hence, the current knowledge on the health consequences of increasing alcohol consumption among older adults is inconsistent.

Self-rated health (SRH) is a subjective measure of the current state of health. SRH has been widely used in population surveys and is a well-known predictor of future health outcomes, use of health services, and mortality in adults over 60 years, even in populations without a known disease burden [28–30], including this study population [31]. Physical illnesses, mental health, sex and social context are related to SRH, especially in older adults [32]. The novelty of using SRH as an outcome indicator for the health consequences of alcohol consumption is its ease of use because it only consists of a single question, and its ability to predict the use of health services and health expenditures [33, 34]. Evidence suggests that SRH captures a wide range of health dimensions, including physical, psychological, and functional health [28, 31]. Understanding the mechanisms for maintaining good SRH in aging in all sexes, as well as risk factors for poorer SRH, can identify opportunities for health promotion and interventions [29, 30].

Although the relationship between alcohol consumption and mortality in older adults has been investigated to some extent, the findings are inconsistent due to the use of different alcohol measures (e.g., average consumption, accumulated consumption, frequency of consumption, binge drinking), few studies with repeated measures on alcohol consumption introducing reversed causality (sick quitters), and weak adjustment for confounders [9, 11, 14, 15, 20, 24]. Findings are also inconsistent on whether women and men have differing mortality risks from the same levels of alcohol use, some indicating that older women tolerate alcohol as well as older men [16, 24, 30].

SRH is an interesting outcome measure, but it can also be an important confounder, which may affect both all-cause death and participants’ adjustment of drinking levels according to self-perceived health. Therefore, we wanted to investigate both outcome variables in the same study. To the best of our knowledge, no studies have longitudinally examined the relationship between alcohol and the health-related consequences measured by SRH and mortality in a population of older adults who have been shown to have increased frequent alcohol consumption four- to eightfold over the past 20 years [4].

### Study aims

I. Investigate the longitudinal relationship between alcohol consumption, self-rated health (SRH) and all-cause mortality risk in a general population of older adults (aged 60 years or older), with adjustment for potential confounders.
II. Examine whether the relationship between alcohol and the health-related outcomes is moderated by possible risk factors.

## Methods

**Fig. 1:**
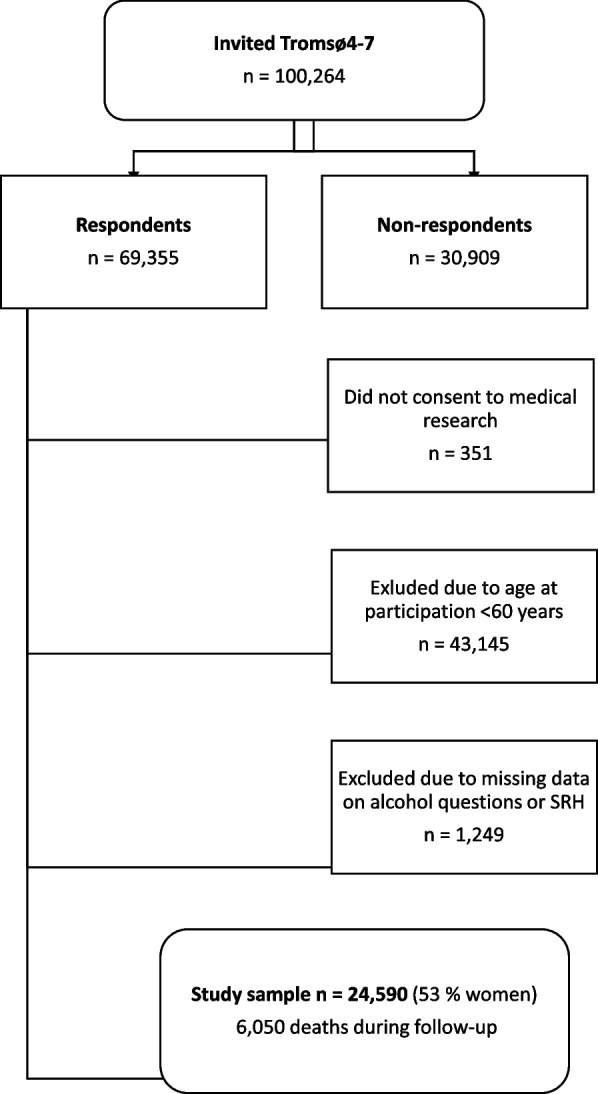
Flow chart of the observations included in the survival analyses from 1994 to 2020. The Tromsø Study

**Fig. 2:**
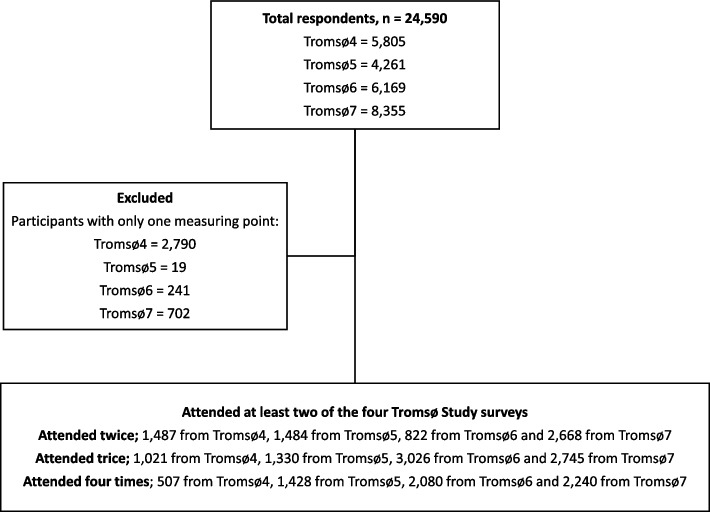
Flow chart for inclusion in the accelerated longitudinal random effects analyses of health trajectories. The Tromsø Study 1994–2016. Each participant was followed for two or more measuring points in the analyses of health trajectories. Thus; the participants could enter the study at different time points, and their first measuring point was regarded as the baseline. Tromsø7 (2015–16) contained the most recent possible observations

This cohort study with repeated cross-sectional examinations was conducted in a general population living in a geographically defined area in Norway. The Tromsø study is an ongoing population-based cohort study conducted in the municipality of Tromsø, and consists of seven surveys (referred to as Tromsø1–7) [35]. The current study is based on the four latest surveys, Tromsø4 (1994–95), Tromsø5 (2001), Tromsø6 (2007–08) and Tromsø7 (2015–16). The overall attendance rates for participants aged 60 years and over were 78, 87, 69, and 68%, respectively, in each consecutive wave. We excluded subjects who had missing values on alcohol consumption questions, leaving 5805 (44 excluded), 4261 (657 excluded), 6169 (291 excluded), and 8355 (261 excluded) participants, from each of the consecutive Tromsø surveys (Fig. 1). Modelling of health trajectories required at least two measuring points and thus included 20,840 observations (Fig. 2). Overall, 6050 deaths were recorded in 15,517 unique participants during the study period.

### Primary outcome measures: self-rated health and all-cause mortality

SRH was the first outcome variable of interest and was measured by the following question: “How do you generally consider your own health?”. The response alternatives were “bad/very bad” (1), “neither good nor bad/fair” (2), “good” (3), and “excellent” (4). Each participant was followed for two or more measuring points in the analyses of health trajectories. Thus; the participants could enter the study at different time points, and their first measuring point was regarded as the baseline. Tromsø7 (2015–16) contained the most recent possible observations.

All-cause mortality was the other outcome of interest. Time of death was retrieved from the Norwegian Cause of Death Registry (CoDR). Follow-up time extended from the date of first participation to the date of death, emigration or the end of study follow-up on November 25, 2020. The coverage of the CoDR is almost complete [36].

### Independent of interest: alcohol consumption

Alcohol consumption was measured with an adaptation of the Alcohol Use Disorders Identification Test-Consumption (AUDIT-C), which is an abbreviated version of the 10-item AUDIT) [37]. The AUDIT-C consists of three questions on the past year’s frequency of drinking (never, monthly or less, 2–4 times a month, 2–3 times a week, or four or more times a week), the number of units consumed on a typical drinking day (1–2, 3–4, 5–6, 7–9, or 10 or more), and frequency of heavy episodic drinking (HED) defined as 6+ units (never, less than monthly, monthly, weekly, daily or almost daily). In Norway, one unit of alcohol is defined as 12 g of pure ethanol. The questions on alcohol consumption differed slightly between the surveys. A comprehensive description of the alcohol consumption measurements and how they were operationalised for comparability is given elsewhere [4]. Abstainers were defined as participants who reported “never” drinking in the previous 12 months or answered “Yes” for teetotallers. The quantity of alcohol was estimated by multiplying the midpoint of each response to AUDIT Item 1 by the midpoint of each response to AUDIT Item 2, thus generating a volume in grams of ethanol per day. Weekly consumption (g/week) was subsequently recoded as a categorical variable with three levels (abstainers, < 100 g/week, and ≥ 100 g/week), as there is some evidence for a low-risk drinking threshold of 100 g/week [14]. Heavy episodic drinking (i.e., 6+ in one sitting) was dichotomised, differentiating between participants with frequent (monthly or more often) or infrequent (less than monthly) binge drinking.

### Covariates

#### Social and demographic variables

Age was measured as a continuous variable and additionally recoded into age groups of 60–64 years, 65–69 years, 70–74 years, and 75 years and older. Educational level was categorised as “primary/elementary school (up to 10 years)” (1), “secondary/upper secondary education (up to an additional three years)” (2), and “college/university/tertiary education (at least four additional years)” (3). Relationship status was assessed by the question “Do you live with a spouse/partner?”, with the response alternatives of “Yes” or “No”. Social support questions were “Do you have enough friends who can give you help and support when you need it?”, and “Do you have enough friends you can talk confidentially with?” with the response alternatives of “Yes” or “No”.

#### Health characteristics

Specially trained personnel measured nonfasting total cholesterol (mmol/l), blood pressure (systolic/diastolic blood pressure, mean of reading 2 and 3) and body weight and height (kg/m^2^). The thresholds for high cholesterol (≥5.0 mmol/l) and high blood pressure (> 140/90 mmHg) were set according to national guidelines for the prevention of cardiovascular disease [38]. Body mass index (BMI) was categorised as “lean,” (< 25 kg/m^2^) “overweight” (25–30 kg/m^2^), or “obese” (≥30 kg/m^2^). Known physical illness were self-reported as specific medical conditions reported in different surveys: psoriasis, food allergies, chronic bronchitis, migraine, ulcer, asthma, thyroid disease, arthritis, myocardial infarction, cerebrovascular stroke, diabetes, osteoporosis, and angina. We used a validated measure of comorbid burden, the Health Impact Index (HII), which considers that each condition has a different impact on SRH [39]. HII was used as a continuous variable in the models and categorised as “Not ill” (0), “Mildly ill” (1, 2), “Moderately ill” (3–5), and “Seriously ill” (≥6) in descriptive statistics. Mental distress was measured with validated questions on degree of anxiety and depression. Tromsø4 used the seven-item Cohort Norway Mental Health Index (CONOR-MHI), whereas Tromsø5–7 used the ten-item Hopkins Symptom Check List-10 (HSCL-10) [40]. The agreement between these questions has been examined with reasonably good compliance [41]. A cut-off of 2.15 for significant symptoms of CONOR-MHI is equivalent to 1.85 for HSCL-10. The suggested cut-off limits were used to estimate an ordinal measure of mental distress: “No symptoms” (0), “Some symptoms” (1), “Subthreshold symptoms” (2), and “Significant symptoms” (3). Self-reported use of sleeping pills or tranquilisers during the last two (Tromsø4) or four (Tromsø5–7) weeks was included (not used, less frequently than every week, every week, but not daily, or daily). The response alternatives were dichotomised as “Have used” or “Have not used” sleeping pills/tranquilisers during the last 2/4 weeks. Data on smoking were measured by the question “How many years in all have you smoked daily?” and were subsequently recoded as “Never” (0), “< 20 years” (1), and “≥20 years” (2). Physical activity level was estimated as an ordinal variable: “inactive” (0), “<1 h/week” (1), “1–2 hours/week” (3), and “3 or more hours/week” (3). High- and low-intensity activity levels were collapsed, and the highest number of hours per week was used.

### Statistics

We performed the statistical analyses in four stages using STATA, version 17.0.

#### Stage 1: descriptive characteristics

We calculated the variables’ means, standard deviations, and percentages according to sex and alcohol consumption. We also performed calculations according to the different surveys to convey information on the changes in characteristics over time.

#### Stage 2: SRH levels across surveys

SHR is not necessarily a stable measure across time. Therefore, we examined mean values of SRH according to age groups versus drinking thresholds for each survey.

#### Stage 3: random-effects models

The multilevel random-effects modelling uses the fact that the data are multiple observations nested in the participants over time. We organised the data as panel data and fitted two-level random-effects logistic models for ordered responses (SRH = bad/fair/good/excellent), with drinking level as the predictor variable and with the time-varying covariates of each panel (i) nested within participants (j) [42]. The referent group for all models were low-risk drinkers (< 100 g/week). We used an accelerated longitudinal design, which includes multiple single trajectories, each starting at a different time relative to the outcome measures. One of the benefits of this method is its ability to span the age range of interest in less time than would be possible with a single cohort longitudinal design [43]. The model combines within- and between-individual effects. The model thus shows the covariance over time, e.g. how SRH changes when alcohol consumption changes adjusted for the other variables. Random effects were used to cope with the potential bias accelerated longitudinal designs have due to multiple cohorts. The method allowed us to adjust for all the independent covariates across surveys. It is reasonable to assume that men and women may adjust alcohol consumption differently according to their perceived health situation as well according to other risk factors. Therefore, we examined which factors affect SRH according to sex. The sex-stratified models were built hierarchically, starting with separate models for each risk factor controlled for age and education. We checked all interactions one by one between each of the risk factors and drinking level in the final model, including the insignificant risk factors, as they may interact with alcohol consumption and affect outcome (SRH), even if they did not reach significance as confounders [44].

#### Stage 4: cox proportional hazards analyses

We estimated the hazard ratios and 95% confidence intervals for death according to alcohol consumption stratified by sex. All time-varying scores were updated in 2001, 2007–08, and 2015–16 for all participants. The models include repeated measures of alcohol consumption to capture the effect of changes in consumption level over time. We followed the same hierarchical analysis plan as in stage 3. Thus, interaction terms between drinking level and all risk factors were examined consecutively in the fully fitted Cox models. Time extended from the age at study entry to the age of death, or end of follow-up on 25 November 2020. The average follow-up time was 11.7 years.

Age, as expected, was strongly correlated with both SRH and mortality, as increasing age reduced SRH and increased the risk of all-cause mortality. A higher educational level was also strongly correlated with better health outcomes (both SRH and mortality) for all levels of alcohol consumption, often referred to as “the alcohol-harm paradox” [45–48]. Thus, all models were corrected by age (continuous) and educational level. The results for age and educational level are not reported as odds ratios (OR) with 95% confidence intervals (CI), as these risk factors are well-known, and not the focus of our study.

## Results

### Characteristics of participants according to drinking habits

The distribution of observations for each alcohol consumption category shows that more women than men abstained from alcohol, while more men consumed ≥100 g/week than women. The average alcohol consumption among participants exceeding 100 g/week was lower in women than men, i.e., 118.8 g/week (SD 48.9 g/week) and 139.0 g/week (SD 74.3 g/week), respectively. Heavy drinkers were younger, had a higher level of education, reported better SRH and fewer illnesses and were more physically active than moderate drinkers and abstainers. Women who reported higher levels of alcohol consumption more often reported having many friends, while female abstainers more often lived alone. More than half of women and men who drank ≥100 g/week reported ≥20 years of daily smoking. Among participants who drank ≥100 g/week, the proportions who reported drinking 6+ units monthly or more often were 39% in men and 14% in women (Table 1).

**Table 1:** Characteristics of the participants ≥60 years according to alcohol consumption in the Tromsø Study 1994–2016

	Abstainers		< 100 g ethanol per week		≥100 g ethanol per week		***P***-value^**e**^
	Women n (%)	Men n (%)	Women n (%)	Men n (%)	Women n (%)	Men n (%)	
**Self-rated health**							
Poor	291 (8.6)	116 (8.0)	405 (4.7)	370 (4.4)	23 (2.7)	80 (5.0)	< 0.001
Fair	1767 (52.2)	619 (42.9)	3296 (37.9)	2989 (35.2)	216 (25.6)	445 (27.6)	
Good	1197 (35.4)	619 (42.9)	4296 (49.3)	4523 (53.2)	449 (53.3)	901 (55.9)	
Excellent	129 (3.8)	89 (6.2)	711 (8.2)	617 (7.3)	155 (18.4)	186 (11.5)	
**Age group**							
60–64 years	635 (18.7)	296 (20.4)	3098 (35.4)	2909 (34.1)	398 (47.0)	656 (40.5)	< 0.001
65–69 years	715 (21.0)	318 (22.0)	2444 (27.9)	2366 (27.8)	232 (27.4)	508 (31.4)	
70–74 years	749 (22.0)	326 (22.5)	1642 (18.8)	1759 (20.6)	122 (14.4)	262 (16.2)	
75 years and older	1302 (38.3)	508 (35.1)	1566 (17.9)	1492 (17.5)	95 (11.2)	192 (11.9)	
Total^†^	3401 (70.1)	1448 (29.9)	8750 (50.6)	8526 (49.4)	1618 (65.6)	847 (34.4)	
**Educational level**							
Elementary school (up to 10 years)	2587 (77.0)	860 (59.9)	4423 (50.9)	3500 (41.3)	160 (19.0)	293 (18.2)	< 0.001
High school (up to an additional three-four years)	513 (15.3)	368 (25.6)	2341 (26.9)	2691 (31.8)	251 (29.8)	465 (28.9)	
College/university, short and long	259 (7.7)	207 (14.4)	1928 (22.2)	2277 (26.9)	431 (51.2)	851 (52.9)	
**Relationship status**							
Live with a spouse or a partner	1527 (52.3)	1027 (77.9)	5103 (62.6)	6714 (81.7)	587 (71.8)	1317 (83.5)	< 0.001
Live alone	1393 (47.7)	291 (22.1)	3047 (37.4)	1506 (18.3)	230 (28.2)	261 (16.5)	
**Enough friends and social support**							
Yes	2536 (85.3)	1106 (86.3)	7487 (89.9)	7243 (89.2)	777 (92.6)	1424 (89.5)	< 0.001
No	436 (14.7)	176 (13.7)	839 (10.1)	880 (10.8)	62 (7.4)	167 (10.5)	
**Average physical activity per week**							
Inactive	794 (24.1)	257 (18.1)	960 (11.2)	840 (10.0)	52 (6.3)	143 (8.9)	< 0.001
< 1 Hour	568 (17.2)	212 (14.9)	1489 (17.4)	1776 (21.1)	121 (14.6)	318 (19.8)	
1–2 hours	898 (27.2)	376 (26.4)	2852 (33.3)	2533 (30.2)	306 (36.8)	465 (29.0)	
≥ 3 hours	1038 (31.5)	577 (40.6)	3269 (38.1)	3251 (38.7)	352 (42.4)	677 (42.2)	
**Health impact index (HII)**^**a**^							
Not ill (HII = 0)	1057 (31.1)	552 (38.1)	3136 (35.8)	4107 (48.2)	379 (44.7)	915 (56.6)	< 0.001
Mildly ill (HII = 1–2)	815 (24.0)	417 (28.8)	2707 (30.9)	2320 (27.2)	284 (33.5)	427 (26.4)	
Moderately ill (HII = 3–5)	894 (26.3)	321 (22.2)	1854 (21.2)	1505 (17.6)	144 (17.0)	216 (13.3)	
Seriously ill (HII ≥ 6)	635 (18.7)	158 (10.9)	1054 (12.0)	594 (7.0)	40 (4.7)	60 (3.7)	
**Body Mass Index**							
Lean (< 25 kg/m2)	1077 (32.0)	481 (33.5)	3286 (37.7)	2594 (30.5)	398 (47.2)	458 (28.3)	< 0.001
Overweight (25–30 kg/m2)	1338 (39.7)	721 (50.2)	3564 (40.9)	4279 (50.3)	339 (40.2)	846 (52.4)	
Obese (≥30 kg/m2)	954 (28.3)	234 (16.3)	1866 (21.4)	1627 (19.1)	107 (12.7)	312 (19.3)	
**Blood pressure**							
< 140/90 mmHg	1082 (31.9)	587 (40.5)	4057 (46.4)	3955 (46.4)	457 (54.1)	727 (45.0)	< 0.001
≥ 140/90 mmHg	2311 (68.1)	861 (59.5)	4681 (53.6)	4563 (53.6)	388 (45.9)	888 (55.0)	
**Total cholesterol**							
< 5.0 mmol/l	425 (12.5)	434 (30.1)	1267 (14.5)	2401 (28.2)	108 (12.8)	433 (26.8)	< 0.001
≥ 5.0 mmol/l	2963 (87.5)	1008 (69.9)	7445 (85.5)	6105 (71.8)	736 (87.2)	1182 (73.2)	
**Never smoked**	1666 (60.2)	393 (29.0)	3051 (37.8)	1959 (23.8)	236 (28.6)	365 (23.1)	< 0.001
> 1–20 years	270 (9.8)	201 (14.8)	1394 (17.3)	1381 (16.8)	159 (19.3)	298 (18.9)	
≥20 years	832 (30.1)	760 (56.2)	3621 (44.9)	4889 (59.4)	429 (52.1)	916 (58.0)	
**Mental distress**^**b**^							
No symptoms	582 (18.2)	413 (29.4)	1935 (23.2)	3010 (36.3)	218 (26.5)	580 (36.1)	< 0.001
Some symptoms	1461 (45.7)	675 (48.1)	3792 (45.5)	3756 (45.2)	361 (43.9)	687 (42.8)	
Sub-threshold symptoms	767 (24.0)	229 (16.3)	1893 (22.7)	1226 (14.8)	169 (20.5)	252 (15.7)	
Significant symptoms	384 (12.0)	86 (6.1)	723 (8.7)	311 (3.7)	75 (9.1)	86 (5.4)	
**Use of sleeping pills/tranquilisers**^**c**^							
Not used last 2/4 weeks	2538 (74.6)	1215 (83.9)	6741 (77.0)	7581 (88.9)	669 (79.0)	1383 (85.5)	< 0.001
Have used last 2/4 weeks	863 (25.4)	233 (16.1)	2009 (23.0)	945 (11.1)	178 (21.0)	235 (14.5)	
**Heavy episodic drinking**^**d**^							
6+ less frequently than monthly	3105 (99.9)	1301 (99.8)	7301 (98.1)	6893 (91.6)	696 (86.0)	937 (61.5)	< 0.001
6+ monthly or more often	4 (0.1)	3 (0.2)	143 (1.9)	634 (8.4)	114 (14.0)	591 (38.7)	
**Average alcohol consumption per week (SD)**	0.0	0.0	13.8 (15.5)	18.2 (17.1)	118.8 (48.9)	139.0 (74.3)	< 0.001

^†^Total = sex distribution in each alcohol consumption category

^a^HII measures physical illness according to the impact that each condition has on SRH

^b^In 1994–95, the seven-item CONOR Mental Health Index (CONOR-MHI) was used, whereas in the three subsequent surveys, the ten-item Hopkins Symptom Check List-10 (HSCL-10) was used

^c^The proportion includes the use of either or both sleeping pills/tranquilisers. In 1994–95, the time frame asked was “during the last 2 weeks”, while in the three subsequent surveys it was “during the last 4 weeks”

^d^Only participants < 70 years were asked the question “how often do you drink 6+ units in one occasion” in 1994–95

^*e*^*P*-values are based on chi square test for all categorical covariates, not stratified by sex: SRH: Pearson chi2 (6) = 719.93, *p* = < 0.000; 5-year age group: Pearson chi2 (6) = 1.3e+ 03, *p* < 0.000; Educational level: Pearson chi2 (4) = 2.4e+ 03, < 0.000; Relationship status: Pearson chi2 (2) = 330.10, *p* < 0.000; Social support: Pearson chi2 (2) = 60.65, < 0.000; Activity level: Pearson chi2 (6) = 516.30, *p* < 0.000; HII group: Pearson chi2 (6) = 547.25, *p* < 0.000; BMI: Pearson chi2 (4) = 69.85, *p* < 0.000; Hypertension: Pearson chi2 (2) = 235.72, *p* < 0.000; Hypercholesterolemia: Pearson chi2 (2) = 31.38, *p* < 0.000; Smoke: Pearson chi2 (4) = 636.11, *p* < 0.000; Mental distress: Pearson chi2 (6) = 208.38, *p* < 0.000; Use of sleeping pills: Pearson chi2 (2) = 80.79, *p* < 0.000; Heavy episodic drinking: Pearson chi2 (2) = 2.4e+ 03, *p* < 0.000. *P*-value for average alcohol consumption (continuous) is based on ANOVA: F (2) = 18,267.32, *p* < 0.000

### Changes in sample characteristics from 1994 to 2016

Alcohol abstention rates were reduced from 31% in 1994–95 to 11% in 2015–16. The proportion who reported alcohol consumption < 100 g/week increased from 65 to 73%, while the proportion who reported exceeding 100 g/week increased from 4 to 16% during the study period. The proportion reporting “good” or “excellent” SRH increased from 45% in 1994–95 to 57% in 2015–16. The educational level increased for each consecutive wave, and the proportion with the highest level of education (college/university) increased from 10% in Tromsø4 to 37% in the latest survey. The proportion of never smokers increased from 23 to 38%. The proportion reporting to be “moderately” (HII = 3–5) or “seriously” (HII = ≥6) ill decreased from 38 to 16%, the proportion with hypertension (≥140/90 mmHg) decreased from 69 to 44%, and the proportion with hypercholesterolemia (≥5.0 mmol/l) decreased from 94 to 69% between Tromsø4 and Tromsø7 (Additional file 1 S. Table 1).

### Overall impact of exceeding 100 g/week of alcohol on self-rated health

We found that a higher level of SRH was reported for each subsequent study for both moderate- and high-level drinkers but not for abstainers (*p* = 0.547). The results from the multilevel random-effects models show that consuming ≥100 g/week was associated with higher levels of SRH in women but not in men (Table 2). This was true in both the univariate and fully adjusted models. In addition, abstaining from alcohol was strongly correlated with poorer SRH in both the univariate and fully adjusted models in women but only modestly in the univariate model in men.

**Table 2:** Results from the random effects models with estimates for the association of subject-specific factors on self-rated health. The Tromsø Study 1994–2016

	Women		Men	
	Univariate OR [95% CI]	Adjusted OR [95% CI]	Univariate OR [95% CI]	Adjusted OR [95% CI]
**Alcohol consumption**				
Abstainer, not consumed alcohol last 12 months	0.49^***^ [0.42, 0.58]	0.60^***^ [0.51, 0.72]	0.76^*^ [0.61, 0.95]	0.85 [0.68, 1.07]
< 100 g ethanol per week	1 (ref.)	1 (ref.)	1 (ref.)	1 (ref.)
≥ 100 g ethanol per week	1.90^***^ [1.49, 2.42]	1.85^***^ [1.46, 2.34]	1.13 [0.94, 1.36]	1.18 [0.99, 1.42]
**Mental distress**^a^				
No symptoms	1 (ref.)	1 (ref.)	1 (ref.)	1 (ref.)
Some symptoms	0.39^***^ [0.34, 0.45]	0.41^***^ [0.35, 0.47]	0.34^***^ [0.30, 0.39]	0.39^***^ [0.34, 0.45]
Sub-threshold symptoms	0.10^***^ [0.09, 0.12]	0.14^***^ [0.12, 0.17]	0.11^***^ [0.09, 0.13]	0.15^***^ [0.12, 0.18]
Significant symptoms	0.03^***^ [0.02, 0.03]	0.05^***^ [0.04, 0.06]	0.02^***^ [0.02, 0.03]	0.04^***^ [0.03, 0.06]
**Physical illness (HII)**^b^	0.75^***^ [0.74, 0.77]	0.77^***^ [0.75, 0.79]	0.72^***^ [0.70, 0.74]	0.74^***^ [0.72, 0.76]
**Smoking**				
Never smoked	1 (ref.)	1 (ref.)	1 (ref.)	1 (ref.)
> 1–20 years	1.11 [0.91, 1.36]	1.05 [0.87, 1.27]	0.73^**^ [0.59, 0.90]	0.74^**^ [0.60, 0.91]
> 20 years	0.61^***^ [0.52, 0.72]	0.70^***^ [0.60, 0.81]	0.35^***^ [0.29, 0.42]	0.46^***^ [0.39, 0.55]
**Have used pills**^**c**^**last 2/4 weeks**	0.42^***^ [0.36, 0.48]	0.69^***^ [0.60, 0.81]	0.36^***^ [0.30, 0.43]	0.70^***^ [0.57, 0.85]
**Body Mass Index**				
Lean (< 25 kg/m2)	1 (ref.)	1 (ref.)	1 (ref.)	1 (ref.)
Overweight (25–30 kg/m2)	0.78^***^ [0.68, 0.90]	0.73^***^ [0.63, 0.84]	0.91 [0.78, 1.06]	0.83^*^ [0.71, 0.97]
Obese (≥30 kg/m2)	0.43^***^ [0.36, 0.52]	0.47^***^ [0.39, 0.56]	0.49^***^ [0.40, 0.59]	0.53^***^ [0.43, 0.64]
**Average physical activity per week**				
Inactive	1 (ref.)	1 (ref.)	1 (ref.)	1 (ref.)
< 1 Hour	1.48^***^ [1.19, 1.84]	1.12 [0.88, 1.41]	1.33^*^ [1.06, 1.68]	1.00 [0.79, 1.27]
1–2 hours	2.27^***^ [1.86, 2.77]	1.49^***^ [1.20, 1.85]	2.12^***^ [1.70, 2.65]	1.50^***^ [1.19, 1.89]
≥ 3 hours	3.51^***^ [2.87, 4.30]	2.25^***^ [1.81, 2.80]	3.01^***^ [2.42, 3.75]	2.10^***^ [1.67, 2.65]
**Social support**	2.77^***^ [2.27, 3.38]	1.53^***^ [1.24, 1.90]	2.14^***^ [1.76, 2.60]	1.35^**^ [1.10, 1.66]

*SRH* self-rated health, *OR* odds ratio, are based on subjects participating ≥ two times with repeated measures of alcohol consumption (*n* = 20,840). All time-varying scores were updated in 2001, 2007–08, and 2015–16 for those who participated. Exponentiated coefficients; 95% confidence intervals in brackets, all estimates are adjusted for education and age

Univariate models: separate models for each risk factor, to estimate the independent effect on the ordinal response variable

Adjusted models: fully fitted models including all listed covariates

^*^p < 0.05

^**^p < 0.01

^***^p < 0.001

^a^In 1994–95, the seven-item CONOR Mental Health Index was used, whereas in the three subsequent surveys, the ten-item Hopkins Symptom Check List-10 (HSCL-10) was used

^b^HII measures physical illness according to the impact that each condition has on SRH

^c^Persons reporting the use of either or both sleeping pills/tranquilisers. In 1994–95, the time frame asked was “during the last 2 weeks”, while in the three subsequent surveys it was “during the last 4 weeks”

### Other factors associated with self-rated health

Mental distress was the strongest independent predictor of poorer SRH in both women and men (Table 2). The second strongest predictor was the use of sleeping pills or tranquilisers. A higher score on the HII (physical illness), ≥20 years of daily smoking, and being obese were individual risk factors predicting poorer SRH. Surprisingly, binge drinking was not associated with poorer SRH. Higher activity levels were increasingly beneficial, and physical activity at 3 h a week or more increased the likelihood of improved SRH by two to three and a half times. Having enough friends/social support was also very beneficial for SRH and increased the odds of better SRH by two to three times.

### Factors that moderate the impact of alcohol consumption on self-rated health

**Fig. 3:**
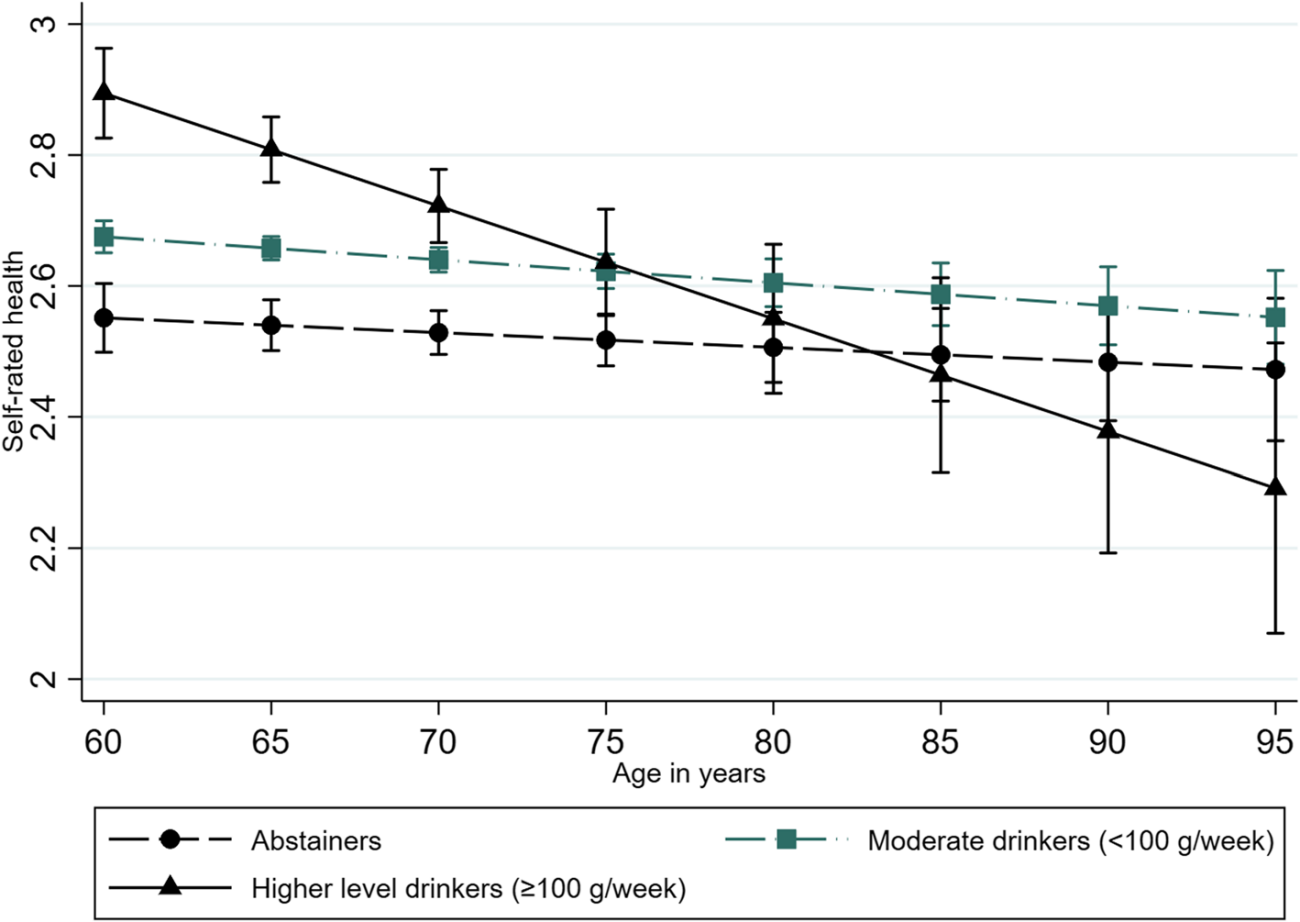
Self-rated health trajectories in women ≥60 years according to alcohol consumption. The Tromsø Study 1994–2016. Results from the postestimation plot based on the fully fitted multilevel random-effects model of self-rated health including the interaction term (alcohol*age) in women (*n* = 10,969). The analysis is based on subjects participating ≥ two times with repeated measures of alcohol consumption

**Fig. 4:**
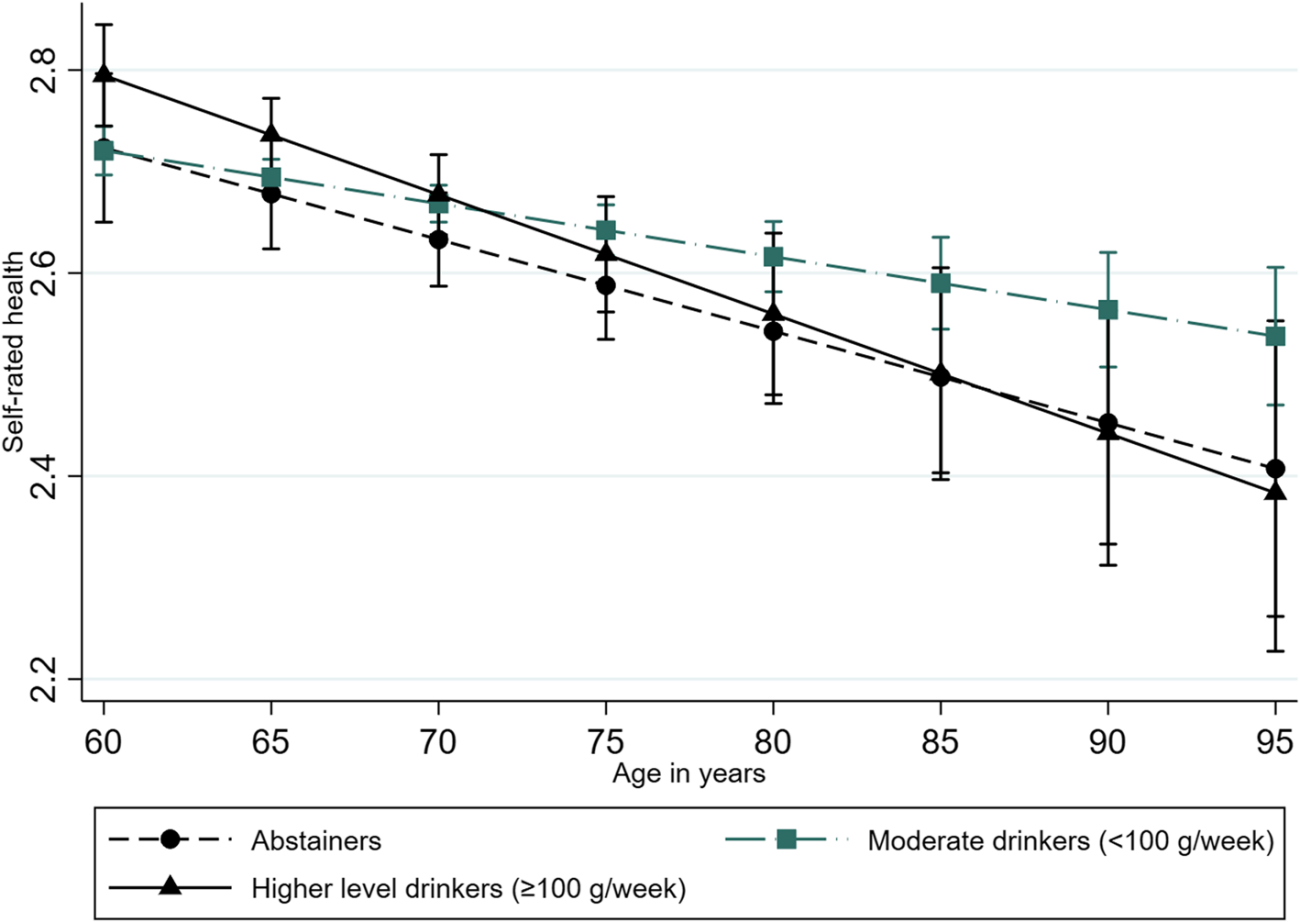
Self-rated health trajectories in men ≥60 years according to alcohol consumption. The Tromsø Study 1994–2016. Results from the postestimation plot based on the fully fitted multilevel random-effects model of self-rated health including the interaction term (alcohol*age) in men (*n* = 9871). The analysis is based on subjects participating ≥ two times with repeated measures of alcohol consumption

We found a significant interaction between alcohol consumption and age in women who exceeded 100 g/week, increasing the odds of reduced SRH by each 10-year period (OR 0.56, 95% CI 0.39–0.82), but this interaction was not significant in men. Figures 3 and 4 show the postestimation plots. SRH declines for all three alcohol categories with increasing age. However, the models predict different trajectories according to sex for different alcohol consumption levels. Women have better SRH when exceeding 100 g/week than moderately drinking women and abstainers up to approximately 75 years of age, while the 95% CIs for the three categories overlap at older ages. The 95% CIs for the three categories overlap at all ages in men, but a steeper decline in SRH with increasing age is observed in men who drink heavily than in men who are abstainers or drink moderately.

The interaction analysis between alcohol and morbidity (HII) was not significant in men exceeding 100 g/week (*p* = 0.561) but was associated with poorer SRH in women (OR 0.87, 95% CI 0.78–0.98). Abstaining from alcohol increased the odds of better SRH for each increase in HII score; OR 1.13 (95% CI 1.04–1.22) in men, and OR 1.07 (95% CI 1.01–1.13) in women. In the interaction term between alcohol consumption and the use of sleeping pills or tranquilisers, exceeding 100 g/week was associated with poorer SRH in men (OR 0.60, 95% CI 0.37–0.99), whereas abstaining while using sleeping pills or tranquilisers was associated with better SRH (OR 2.46, 95% CI 1.33–4.56). This interaction was not significant in women. Interaction testing between alcohol consumption and smoking showed that men who had smoked for ≥20 years in combination and exceeded ≥100 g/week reported poorer SRH (OR 0.61, 95% CI 0.39–0.94). This interaction was not significant for women.

### Overall impact of exceeding 100 g/week of alcohol on all-cause mortality risk

**Fig. 5:**
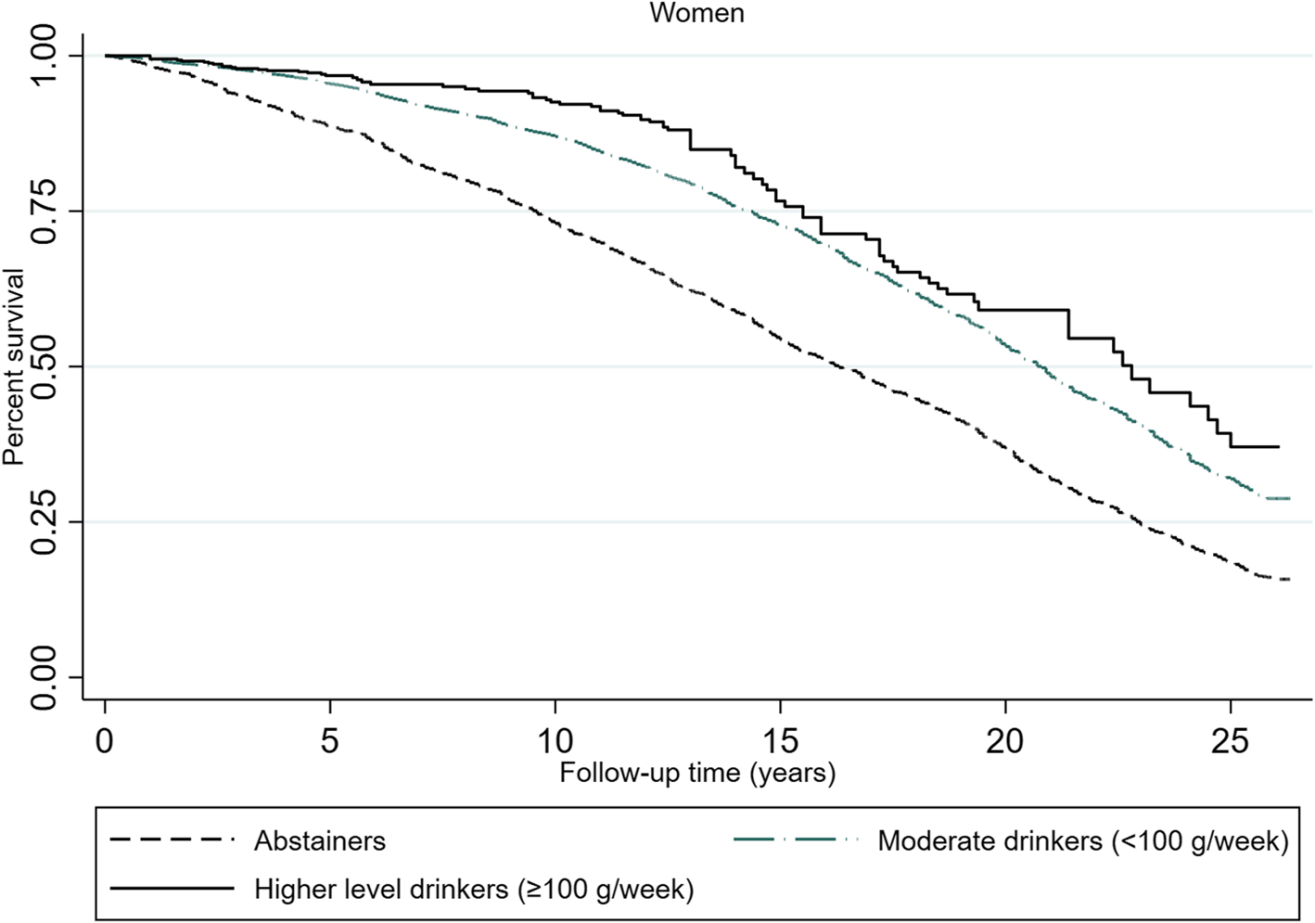
Survival plot according to alcohol consumption level for women ≥60 years. The Tromsø Study 1994–2020. Kaplan-Meier function based on cox proportional hazard models with repeated measures of alcohol consumption. Time extended from the age at study entry to the age of death, or end of follow-up on 25 November 2020. The average follow-up time was 11.7 years

**Fig. 6:**
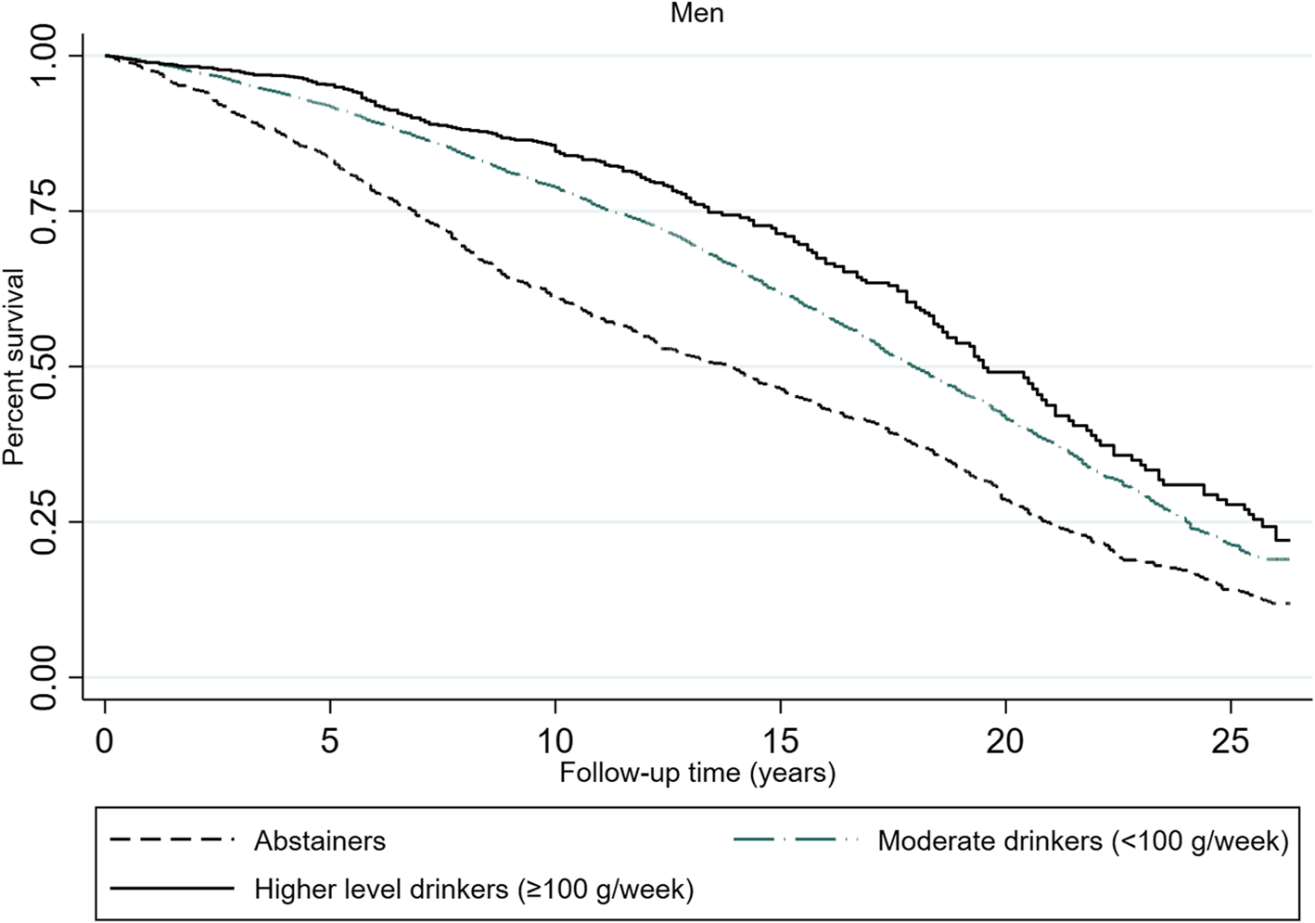
Survival plot according to alcohol consumption level for men ≥60 years. The Tromsø Study 1994–2020. Kaplan-Meier function based on cox proportional hazard models with repeated measures of alcohol consumption. Time extended from the age at study entry to the age of death, or end of follow-up on 25 November 2020. The average follow-up time was 11.7 years

The mortality rates and hazard ratios for the alcohol consumption groups show that women had lower mortality rates than men according to all recorded alcohol consumption levels (Table 3). Abstaining women had almost twice the mortality rate (0.049) as moderately drinking women (0.025) and almost triple the mortality rate as high-level drinking women (0.015). The same pattern was found in men but was not as distinct as that in women. The survival plots show how the mortality risk was most pronounced for abstaining men and women but also that the curve falls more steeply for men than for women (Figs. 5 and 6). The results from the fully fitted Cox models show that the mortality risk was not increased in either women or men who consumed ≥100 g/week compared to those who consumed < 100 g/week (Table 4). Abstinence was associated with 31% increased mortality risk in women (HR 1.31, 95% CI 1.18–1.46) and 18% in men (HR 1.18, 95% CI 1.06–1.32) relative to those who consumed < 100 g/week. The mortality risk was attenuated when controlling for the covariates.

**Table 3:** Mortality rates according to alcohol consumption in subjects aged ≥60 years. The Tromsø Study 1994–2020

	Person Time (Years)	Mortality rate	Number of subjects	Survival time (Years)		
				25%	50%	75%
Female abstainer	28,174	0.0486	2348	9.5	16.3	23.0
Male abstainer	10,268	0.0595	1010	6.9	13.8	21.0
Female moderate drinker (< 100 g/week)	66,260	0.0253	5827	14.4	20.7	.
Male moderate drinker (< 100 g/week)	59,667	0.0346	5615	11.2	18.0	24.0
Female high-level drinker (≥100 g/week)	5517	0.0152	700	15.5	22.8	.
Male high-level drinker (≥100 g/week)	10,499	0.0232	1297	13.4	19.6	25.7
Total	180,384	0.0335	15,517	12.0	18.9	24.8

**Table 4:** All-cause mortality risk by alcohol consumption in subjects aged ≥60 years. The Tromsø Study 1994–2020

	Women		Men	
	Univariate HR [95% CI]	Adjusted HR [95% CI]	Univariate HR [95% CI]	Adjusted HR [95% CI]
**Alcohol consumption**				
Abstainer, not consumed alcohol last 12 months	1.53*** [1.42, 1.65]	1.31*** [1.18, 1.46]	1.37*** [1.25, 1.50]	1.18** [1.06, 1.32]
< 100 g ethanol per week	1 (ref.)	1 (ref.)	1 (ref.)	1 (ref.)
≥ 100 g ethanol per week	0.89 [0.72, 1.11]	0.95 [0.73, 1.22]	0.89 [0.77, 1.01]	0.89 [0.77, 1.03]
**Self-rated health**				
Poor	1 (ref.)	1 (ref.)	1 (ref.)	1 (ref.)
Fair	0.75*** [0.66, 0.85]	0.86 [0.72, 1.02]	0.63*** [0.55, 0.71]	0.71*** [0.61, 0.83]
Good	0.50*** [0.44, 0.56]	0.61*** [0.50, 0.74]	0.40*** [0.35, 0.45]	0.55*** [0.46, 0.64]
Excellent	0.32*** [0.25, 0.41]	0.38*** [0.27, 0.53]	0.25*** [0.20, 0.31]	0.37*** [0.28, 0.49]
**Live with a spouse or a partner**	0.71*** [0.66, 0.77]	0.81*** [0.74, 0.89]	0.77*** [0.70, 0.84]	0.81*** [0.74, 0.90]
**Mental distress**^a^				
No symptoms	1 (ref.)	1 (ref.)	1 (ref.)	1 (ref.)
Some symptoms	1.54*** [1.38, 1.73]	1.17* [1.02, 1.33]	1.68*** [1.53, 1.84]	1.31*** [1.18, 1.44]
Sub-threshold symptoms	1.55*** [1.37, 1.75]	1.07 [0.91, 1.24]	1.85*** [1.65, 2.08]	1.25*** [1.10, 1.43]
Significant symptoms	1.88*** [1.63, 2.18]	1.04 [0.86, 1.26]	2.31*** [1.96, 2.73]	1.36** [1.12, 1.66]
**Physical illness (HII)**^b^	1.05*** [1.04, 1.07]	1.05*** [1.03, 1.06]	1.08*** [1.07, 1.10]	1.07*** [1.05, 1.08]
**Smoking**				
Never smoked	1 (ref.)	1 (ref.)	1 (ref.)	1 (ref.)
1–20 years	1.29*** [1.11, 1.49]	1.12 [0.95, 1.32]	1.35*** [1.15, 1.59]	1.21* [1.03, 1.44]
> 20 years	1.95*** [1.78, 2.14]	1.67*** [1.50, 1.86]	2.49*** [2.21, 2.82]	1.97*** [1.73, 2.24]
**Have used pills**^**c**^**last 2/4 weeks**	0.66*** [0.60, 0.72]	0.78*** [0.70, 0.87]	1.02 [0.92, 1.14]	0.89 [0.80, 1.00]
**High blood pressure (> 140/90 mmHg)**	1.34*** [1.23, 1.45]	1.15** [1.04, 1.28]	1.25*** [1.16, 1.35]	1.26*** [1.16, 1.38]
**Body Mass Index**				
Lean (< 25 kg/m2)	1 (ref.)	1 (ref.)	1 (ref.)	1 (ref.)
Overweight (25–30 kg/m2)	0.72*** [0.66, 0.78]	0.65*** [0.59, 0.73]	0.73*** [0.68, 0.79]	0.73*** [0.67, 0.80]
Obese (≥30 kg/m2)	0.76*** [0.69, 0.84]	0.67*** [0.59, 0.76]	0.68*** [0.61, 0.76]	0.64*** [0.57, 0.73]
**Average physical activity per week**				
Inactive	1 (ref.)	1 (ref.)	1 (ref.)	1 (ref.)
< 1 Hour	0.61*** [0.54, 0.68]	0.71*** [0.61, 0.83]	0.57*** [0.51, 0.65]	0.69*** [0.60, 0.79]
1–2 hours	0.57*** [0.51, 0.62]	0.73*** [0.63, 0.83]	0.58*** [0.52, 0.65]	0.68*** [0.60, 0.78]
≥ 3 hours	0.59*** [0.54, 0.65]	0.81** [0.71, 0.93]	0.63*** [0.57, 0.70]	0.74*** [0.66, 0.84]

*HR* hazard ratios, are based on cox proportional hazard models with repeated measures of alcohol consumption (*n* = 24,590). All time-varying scores were updated in 2001, 2007–08, and 2015–16 for those who participated. End of follow-up on November 25, 2020. Exponentiated coefficients; 95% confi’dence intervals in brackets, all estimates are adjusted for education and age

Univariate models: separate models for each risk factor, to estimate the independent effect on HR

Adjusted models: fully fitted models including all listed covariates

^*^p < 0.05

^**^p < 0.01

^***^p < 0.001

^a^In 1994–95, the seven-item CONOR Mental Health Index (CONOR-MHI) was used, whereas in the three subsequent surveys, the ten-item Hopkins Symptom Check List-10 (HSCL-10) was used

^b^HII measures physical illness according to the impact that each condition has on SRH. ^c^Subjects reporting the use of either or both sleeping pills/tranquilisers. In 1994–95, the time frame asked was “during the last 2 weeks”, while in the three subsequent surveys it was “during the last 4 weeks”

^c^Subjects reporting the use of either or both sleeping pills/tranquilisers. In 1994–95, the time frame asked was “during the last 2 weeks”, while in the three subsequent surveys it was “during the last 4 weeks”

### Other factors associated with all-cause mortality risk

As expected, there was a strong relationship between SRH and all-cause mortality risk (Table 4). A better SRH level (“good” or “excellent”) reduced the hazard ratio by 50–75%. Daily smoking ≥20 years, mental distress, physical illness, and hypertension were independently associated with increased mortality risk. Binge drinking was not related to increased mortality risk. Living with a spouse or a partner, being overweight or obese, and having a higher level of physical activity were independent factors associated with decreased mortality risk. Use of sleeping pills or tranquilisers was associated with a reduced mortality risk in women but not in men.

### Factors that moderate the impact of alcohol consumption on all-cause mortality risk

We found no significant interactions between alcohol consumption and any of the listed covariates on mortality risk.

### Sensitivity analyses

Similar hazard ratio results were obtained when we excluded participants who died within the first year after inclusion to control for those who were already sick. In these analyses, the number of participants was reduced from 15,117 to 13,922 (52% women). The results from the fully fitted models only differed at the second decimal places and beyond. We also repeated our analyses separately for participants born before and after 1946 to compare the effect of exceeding 100 g/week in Baby Boomers, who have increased their alcohol consumption, with the “dry” Pre-War II generation. The results were qualitatively identical in the two cohorts, although the premature mortality risk in the abstaining Baby Boom women was more significant than in the Pre-War II women (Additional file 2 S. Table 2). A wider CI band in mortality risk by alcohol consumption indicates greater heterogeneity in the Baby Boom cohort than in the Pre-War II cohort, possibly due to biases introduced by shorter follow-up time. However, we cannot rule out that this difference implies a change in mortality risk due to changed alcohol habits in the new cohort of older adults.

## Discussion

In this cohort study with up to 25 years of follow-up, we found no clear evidence of an independent negative effect of exceeding an average intake of 100 g of alcohol per week on either SRH or mortality risk when compared with moderate drinking levels in community-dwelling older adults. However, we identified sex-specific differences in the association between alcohol consumption and SRH. A strong positive correlation between a high alcohol consumption and better SRH and a negative correlation between abstaining from alcohol and poorer SRH were identified in women but not in men. The positive relationship between high alcohol consumption and better SRH in women weakened with increasing age. Furthermore, some differences between men and women in risk factors that moderated the relationship between alcohol consumption and SRH were identified.

The positive correlation between the highest level of alcohol consumption and SRH in women was surprising. However, some evidence indicates that alcohol consumption may carry some health benefits for older women in terms of survival and quality of life, possibly mediated through a healthier drinking pattern than men and cardio-protective effects [16, 49–51]. On the other hand, some studies have found that having a very good health status is a predictor of alcohol consumption, and not the other way around [52, 53]. Our finding indicates that frequent drinking in old age is an indicator rather than a cause of the health status, and this is especially the case in women. Furthermore, our finding of a strong correlation between abstinence and poorer SRH in women, but not in men, indicates that the “sick quitter effect” applies especially to older women. Thus, the majority of older women appear to drink according to their health situation, while older men exceed at-risk drinking thresholds regardless of good or poor health. This is in line with other findings of gender differences regarding risky health behaviours, and might explain why possible health benefits are gender specific [23, 54–60].

Even if we found no independent relationship between alcohol consumption and SRH in men, we found that sleeping pills or tranquilisers increased the adverse effect of high alcohol consumption on SRH. This finding concurs with other findings of an increased risk of alcohol problems or increased mortality among older men who report the use of sleeping pills or drugs with addiction potential [61–63]. Moreover, a bidirectional association between sleeping problems and high alcohol consumption has been reported in men but not in women [63]. This implies that a subgroup of men who are prescribed sleeping pills or tranquilisers are at increased risk of a negative impact of alcohol on SRH.

In contrast to other findings that binge drinking is particularly harmful in older adults [8, 12, 64, 65], our study did not find that frequent binge drinking was a significant confounder or moderator for either SRH or all-cause mortality. Others have reported similar findings, which may imply that binge drinking is an imprecise measure to identify harmful uses of alcohol [66, 67]. Having enough friends and social support and a higher activity level were independent beneficial factors for SRH in both sexes. A larger proportion of female heavy drinkers reported being socially satisfied and more physically active than male heavy drinkers. Although there was a lack of clear evidence for a moderating effect, these factors may have mediated a beneficial effect of high alcohol consumption in some women. Our findings may indicate that women and men adjust the risk factors differently so that women maintain better SRH even if they exceed the low-risk drinking thresholds.

The average SRH level improved during the study period. Furthermore, the proportion who had never smoked increased, the proportion with severe physical illness decreased, and the proportion with hypertension or hypercholesterolemia decreased. These findings indicate a healthier elderly population and may have reduced any adverse effects of increased alcohol consumption. In addition, average weekly alcohol consumption, even in the highest consumption group, was just above the threshold in women and not very high in men. This observation can be related to the fact that older adults in high-income western European countries, including Norway, drink level-headedly, i.e., they drink more frequently but consume relatively small amounts of alcohol on each occasion [1, 3, 4, 61]. Moreover, we observed that a higher proportion among the high-level drinkers was highly educated, lean (women), had normal blood pressure (women), had less physical illness, and reported more hours of weekly physical activity. This suggests that a large proportion of older drinkers’ balanced risk factors are beneficial, which is in line with other findings [52, 68, 69].

Recent Canadian guidelines on the prevention, assessment, and treatment of alcohol use disorder recommend that older women drink no more than five alcoholic drinks per week and older men drink no more than seven per week [70]. Contrary to several other countries, Norway do not have sex- and older adult-specific recommendations on drinking thresholds [65, 71, 72]. However, over the last century, Norway has possibly had one of the most restrictive alcohol policies in Europe and among the lowest alcohol per capita consumption (APC) [18, 73]. Strongly influenced by Skog’s theory of the collective components in drinking habits, alcohol sales in Norway are strictly regulated, have limited availability through designated stores, and are relatively expensive due to high taxes [74]. Recent evidence advocates that universal policies targeting APC have the most significant impact on public health, as they are likely an efficient way to prevent people from becoming very heavy drinkers [67, 75]. Our findings do not support the assertion that most older adults need lower limits of regular alcohol use than their younger counterparts, which is in line with other research [22, 24, 62].

### Strengths and limitations

Important strengths of the Tromsø study are the high number of participants and the high proportion of attendance, which ensure that the results are representative of the general population. However, the rates of attendance in the oldest age groups were lower than those in the younger age groups and may therefore be less representative. It was probably healthier subjects who participated, which may have biased the results toward participants who tolerated alcohol better. Furthermore, the participation rate in the Tromsø Study has declined in the consecutive surveys, which may have led to further bias [35]. Excessive alcohol use, abstention from alcohol, and mental distress correlate with nonparticipation [76]. Thus, possible underrepresentation of older adults with excessive alcohol consumption and poor mental health requires caution when interpreting the results from our study. Although self-reporting is a common approach for gathering data in epidemiologic research, bias can arise from social desirability, recall period, sampling approach, selective recall and possible gender reporting differences [77–79]. Moreover, a cohort effect has been found in the importance of mental well-being on SRH, with increasing importance across cohorts [32, 80]. Albeit misclassification bias is important to appraise when conclusions are to be drawn from this study, information bias is assumed to have contributed only to a small extent to reducing the validity of our results.

The methodology of multilevel random-effects analysis is robust [42]. The sample size is large, and thus the power is strong. However, modelling of health trajectories required at least two measurements, which may have further biased our findings towards healthier participants. Nevertheless, the methodology ensures that data are not wasted for participants and occasions for which either the response or the covariates are missing, in contrast to more old-fashioned approaches such as listwise deletion or complete case analysis. Use of all available data is less susceptible to bias [42].

Furthermore, the abstinence group most likely consists of both lifelong, past and current abstainers, introducing problems with reversed causality known as the sick quitters’ bias.

The data retrieved from the Tromsø study are based on citizens living in the seventh-largest Norwegian city, and with relatively few immigrants, the findings are therefore limited concerning ethnic diversity. Furthermore, Norwegian older adults have greater financial security, better health and welfare systems, and less social inequality than in many other European countries. Therefore, the generalisability of the results may be limited to Caucasian populations living in high-income western European countries.

## Conclusion

In the present study, mortality risk in older adults who exceeded 100 g/week of alcohol was not increased compared to those who consumed less than 100 g/week over a 25-year follow-up period. Furthermore, exceeding 100 g/week showed no negative effect on SRH compared with moderate drinking. Our findings indicate that older people who experience poorer health reduce their alcohol consumption, and this applies particularly to women. Since we used updated measurements, it can be assumed that alcohol consumption was adjusted according to increasing health challenges. This may help to explain that we did not find a negative longitudinal relationship between high alcohol consumption in old age and health outcomes. However, some risk factors were linked with reduced SRH and increased mortality risk. We recommend attention to older adults with high-level alcohol consumption who are mentally distressed, have physical illness, report poor SRH, have hypertension, live alone, have smoked for many years or are inactive. Older men with high levels of alcohol consumption who are also prescribed sleeping pills or tranquilisers have an increased risk of adverse health consequences. Our study does not support sex- and older adult-specific recommendations for drinking thresholds in a general population of older adults, but the assumption of a protective effect of drinking on mortality while ignoring the dynamic relationship between poor health and drinking behaviour is probably ill-founded.

## Supplementary Information

**Additional file 1: Table S1.** Characteristics of the participants ≥60 years according to survey. The Tromsø Study 1994–2016. ^a^Only participants < 70 years were asked the question “how often do you drink 6+ units in one occasion” in 1994–95. ^b^The proportion includes the use of either or both sleeping pills/tranquilisers. In 1994–95, the time frame asked was “during the last *two* weeks”, while in the three subsequent surveys it was “during the last *four* weeks”. ^c^HII measures somatic diseases according to the impact that each condition has on SRH. ^d^In 1994–95, the seven-item CONOR Mental Health Index (CONOR-MHI) was used, whereas in the three subsequent surveys, the ten-item Hopkins Symptom Check List-10 (HSCL-10) was used.

**Additional file 2: Table S2.** All-cause mortality risk by alcohol consumption according to cohort. The Tromsø Study 1994–2020. HR; hazard ratios, are based on cox proportional hazard models with repeated measures of alcohol consumption. All time-varying scores were updated in 2001, 2007–08, and 2015–16 for those who participated. All estimates are adjusted for education, age and including all listed covariates. End of follow-up on November 25, 2020. Exponentiated coefficients; 95% confidence intervals in brackets. ^*^p <  0.05, ^**^p <  0.01, ^***^p <  0.001. ^a^In 1994–95, the seven-item CONOR Mental Health Index (CONOR-MHI) was used, whereas in the three subsequent surveys, the ten-item Hopkins Symptom Check List-10 (HSCL-10) was used ^b^HII measures somatic diseases according to the impact that each condition has on SRH. ^c^Subjects reporting the use of either or both sleeping pills/tranquilisers. In 1994–95, the time frame asked was “during the last *two* weeks”, while in the three subsequent surveys it was “during the last four weeks”. ^c^Subjects reporting the use of either or both sleeping pills/tranquilisers. In 1994–95, the time frame asked was “during the last 2 weeks”, while in the three subsequent surveys it was “during the last 4 weeks”.

## Data Availability

The legal restriction on data availability are set by the Tromsø Study Data and Publication Committee in order to control for data sharing, including publication of datasets with the potential of reverse identification of de-identified sensitive participant information. We have received administrative permission to access and use the data that support the findings of this study. A detailed overview of the data collection process, including links to the main questionnaires, can be found on the website of the Tromsø Study (https://uit.no/research/tromsostudy). Data may be obtained from a third party and are not publicly available.

## Abbreviations

CI: Confidence interval
CONOR-MHI: Cohort Norway Mental Health Index
OR: Odds ratio
HED: Heavy episodic drinking (i.e., 6+ in one sitting)
HII: Health impact index
HSCL-10: Hopkins Symptom Check List-10
SRH: Self-rated health
