# Supplementary material for: The effects of exceeding low-risk drinking thresholds on self-rated health and all-cause mortality in older adults: the Tromsø study 1994–2020
Source: Arch Public Health. 2023 Feb 16;81:25. doi: 10.1186/s13690-023-01035-0 (PMC9933408; doi:10.1186/s13690-023-01035-0)
Supplement: Supplementary file 1 — Additional file 1: Table S1. Characteristics of the participants ≥60 years according to survey. The Tromsø Study 1994–2016. aOnly participants < 70 years were asked the question “how often do you drink 6+ units in one occasion” in 1994–95. bThe proportion includes the use of either or both sleeping pills/tranquilisers. In 1994–95, the time frame asked was “during the last two weeks”, while in the three subsequent surveys it was “during the last four weeks”. cHII measures somatic diseases according to the impact that each condition has on SRH. dIn 1994–95, the seven-item CONOR Mental Health Index (CONOR-MHI) was used, whereas in the three subsequent surveys, the ten-item Hopkins Symptom Check List-10 (HSCL-10) was used. [file 13690_2023_1035_MOESM1_ESM.docx]

**S. Table 1** Characteristics of the participants ≥60 years according to survey. The Tromsø Study (1994-2016)

|  |  | **1994-95** | **2001** | **2007-08** | **2015-16** | **Total** |
| --- | --- | --- | --- | --- | --- | --- |
|  |  | n (%) | n (%) | n (%) | n (%) | n (%) |
| **Age, sex and educational level** |  |  |  |  |  |  |
| 60-64 years |  | 1,554 (26.8) | 1,346 (31.6) | 2,396 (38.8) | 2,696 (32.3) | 7,992 (32.5) |
| 65-69 years |  | 1,525 (26.3) | 1,080 (25.3) | 1,594 (25.8) | 2,384 (28.5) | 6,583 (26.8) |
| 70-74 years |  | 1,260 (21.7) | 927 (21.8) | 1,016 (16.5) | 1,657 (19.8) | 4,860 (19.8) |
| 75 years and older |  | 1,466 (25.2) | 908 (21.3) | 1,163 (18.8) | 1,618 (19.4) | 5,155 (21.0) |
| Women |  | 3,212 (55.3) | 2,260 (53.0) | 3,237 (52.5) | 4,289 (51.3) | 12,998 (52.9) |
| Men |  | 2,593 (44.7) | 2,001 (47.0) | 2,932 (47.5) | 4,066 (48.7) | 11,592 (47.1) |
| Elementary school (up to 10 years) |  | 3,729 (64.7) | 2,575 (61.0) | 2,480 (40.5) | 3,039 (36.6) | 11,823 (48.4) |
| High school (up to an additional three-four years) |  | 1,462 (25.4) | 986 (23.3) | 1,969 (32.1) | 2,212 (26.7) | 6,629 (27.2) |
| College/university, short and long |  | 569 (9.9) | 663 (15.7) | 1,679 (27.4) | 3,042 (36.7) | 5,953 (24.4) |
| **Alcohol consumption, use of sleeping pills/tranquilisers** |  |  |  |  |  |  |
| Abstainer, not consumed alcohol last 12 months |  | 1,785 (30.7) | 1,046 (24.5) | 1,075 (17.4) | 943 (11.3) | 4,849 (19.7) |
| >0<100 grams ethanol per week,  (mean (SD)) |  | 3,767 (64.9)  (13.4 (16.3)) | 2,979 (69.9)  (13.0 (14.7)) | 4,451 (72.1)  (16.2 (16.2)) | 6,082 (72.8)  19.1 (16.9) | 17,279 (70.3) |
| ≥100 grams ethanol per week,  (mean (SD)) |  | 254 (4.4)  (131.9 (54.6)) | 237 (5.6)  (131.7 (63.0)) | 644 (10.4)  (130.9 (66.3)) | 1,330 (15.9)  (130.1 (63.0)) | 2,465 (10.0) |
| 6+ less frequently than monthly^a^ |  | 3,363 (90.7) | 3,854 (96.5) | 5,568 (94.0) | 7,453 (92.0) | 20,238 (93.1) |
| 6+ monthly or more often^a^ |  | 343 (9.3) | 141 (3.5) | 356 (6.0) | 649 (8.0) | 1,489 (6.9) |
| Have used pills^b^ last 2/4 weeks |  | 319 (5.5) | 1,139 (26.7) | 1,299 (21.1) | 1,706 (20.4) | 4,463 (18.1) |
| Not used pills^b^ last 2/4 weeks |  | 5,486 (94.5) | 3,122 (73.3) | 4,870 (78.9) | 6,649 (79.6) | 20,127 (81.9) |
| **Self-rated health** |  |  |  |  |  |  |
| Poor |  | 333 (5.7) | 119 (2.8) | 388 (6.3) | 445 (5.3) | 1,285 (5.2) |
| Fair |  | 2,857 (49.3) | 1,756 (41.5) | 2,133 (34.7) | 2,586 (31.1) | 9,332 (38.1) |
| Good |  | 2,398 (41.4) | 2,125 (50.3) | 3,050 (49.7) | 4,412 (53.0) | 11,985 (48.9) |
| Excellent |  | 209 (3.6) | 227 (5.4) | 570 (9.3) | 881 (10.6) | 1,887 (7.7) |
| **Social support, relationship status** |  |  |  |  |  |  |
| Live with a spouse or a partner |  | 3,167 (69.1) | 2,920 (69.4) | 4,290 (70.7) | 5,898 (72.4) | 16,275 (70.8) |
| Live alone |  | 1,415 (30.9) | 1,285 (30.6) | 1,779 (29.3) | 2,249 (27.6) | 6,728 (29.2) |
| Enough friends and social support |  |  |  |  |  |  |
| Yes |  | 3,899 (84.3) | 3,693 (92.6) | 5,386 (87.3) | 7,595 (90.9) | 20,573 (88.9) |
| No |  | 724 (15.7) | 293 (7.4) | 783 (12.7) | 760 (9.1) | 2,560 (11.1) |
| **Average physical activity per week** |  |  |  |  |  |  |
| Inactive |  | 1,123 (19.4) | 394 (9.4) | 887 (14.9) | 642 (7.8) | 3,046 (12.6) |
| <1 Hour |  | 673 (11.6) | 443 (10.6) | 1,489 (25.0) | 1,879 (22.9) | 4,484 (18.6) |
| 1-2 hours |  | 1,548 (26.7) | 1,334 (31.8) | 1,850 (31.1) | 2,698 (32.9) | 7,430 (30.8) |
| ≥3 hours |  | 2,448 (42.3) | 2,022 (48.2) | 1,723 (29.0) | 2,971 (36.3) | 9,164 (38.0) |
| **Daily smokers** |  |  |  |  |  |  |
| Never smoked |  | 1,055 (22.9) | 1,444 (34.6) | 2,162 (36.3) | 3,009 (37.2) | 7,670 (33.6) |
| >1-20 years |  | 555 (12.0) | 626 (15.0) | 1,058 (17.8) | 1,464 (18.1) | 3,703 (16.2) |
| >20 years |  | 2,997 (65.1) | 2,107 (50.4) | 2,733 (45.9) | 3,610 (44.7) | 11,447 (50.2) |
| **Physical illness and metabolic risk factors** |  |  |  |  |  |  |
| Health impact index (HII)^c^ |  |  |  |  |  |  |
| Not ill (HII=0) |  | 2,218 (38.2) | 1,365 (32.0) | 2,283 (37.0) | 4,280 (51.2) | 10,146 (41.3) |
| Mildly ill (HII=1-2) |  | 1,370 (23.6) | 1,125 (26.4) | 1,792 (29.0) | 2,683 (32.1) | 6,970 (28.3) |
| Moderately ill (HII=3-5) |  | 1,298 (22.4) | 980 (23.0) | 1,472 (23.9) | 1,183 (14.2) | 4,933 (20.1) |
| Seriously ill (HII≥6) |  | 919 (15.8) | 791 (18.6) | 622 (10.1) | 209 (2.5) | 2,541 (10.3) |
| Body Mass Index |  |  |  |  |  |  |
| Lean (<25 kg/m2) |  | 2,347 (40.6) | 1,451 (34.1) | 1,930 (31.3) | 2,575 (30.9) | 8,303 (33.9) |
| Overweight (25-30 kg/m2) |  | 2,504 (43.3) | 1,932 (45.5) | 2,872 (46.6) | 3,800 (45.6) | 11,108 (45.3) |
| Obese (≥30 kg/m2) |  | 928 (16.1) | 866 (20.4) | 1,362 (22.1) | 1,958 (23.5) | 5,114 (20.9) |
| Blood pressure |  |  |  |  |  |  |
| < 140/90 mmHg |  | 1,809 (31.2) | 1,742 (40.9) | 2,608 (42.3) | 4,706 (56.4) | 10,865 (44.2) |
| ≥ 140/90 mmHg |  | 3,994 (68.8) | 2,516 (59.1) | 3,551 (57.7) | 3,631 (43.6) | 13,692 (55.8) |
| Total cholesterol |  |  |  |  |  |  |
| < 5.0 mmol/l |  | 379 (6.5) | 527 (12.4) | 1,551 (25.3) | 2,611 (31.4) | 5,068 (20.7) |
| ≥ 5.0 mmol/l |  | 5,410 (93.5) | 3,724 (87.6) | 4,589 (74.7) | 5,716 (68.6) | 19,439 (79.3) |
| **Mental distress**^d^ |  |  |  |  |  |  |
| No symptoms |  | 414 (7.6) | 1,390 (34.5) | 1,973 (33.2) | 2,961 (36.0) | 6,738 (28.5) |
| Some symptoms |  | 3,431 (62.7) | 1,710 (42.4) | 2,411 (40.6) | 3,180 (38.7) | 10,732 (45.3) |
| Sub-threshold symptoms |  | 1,177 (21.5) | 683 (17.0) | 1,049 (18.4) | 1,527 (19.1) | 4,340 (19.0) |
| Significant symptoms |  | 454 (8.3) | 246 (6.1) | 461 (7.8) | 504 (6.1) | 1,665 (7.0) |

^a^Only participants <70 years were asked the question “how often do you drink 6+ units in one occasion” in 1994-95.

^b^The proportion includes the use of either or both sleeping pills/tranquilisers. In 1994-95, the time frame asked was “during the last *two* weeks”, while in the three subsequent surveys it was “during the last *four* weeks”.

^c^HII measures physical illness according to the impact that each condition has on SRH.

^d^In 1994-95, the seven-item CONOR Mental Health Index (CONOR-MHI) was used, whereas in the three subsequent surveys, the ten-item Hopkins Symptom Check List-10 (HSCL-10) was used
