# Supplementary material for: The effects of exceeding low-risk drinking thresholds on self-rated health and all-cause mortality in older adults: the Tromsø study 1994–2020
Source: Arch Public Health. 2023 Feb 16;81:25. doi: 10.1186/s13690-023-01035-0 (PMC9933408; doi:10.1186/s13690-023-01035-0)
Supplement: Supplementary file 2 — Additional file 2: Table S2. All-cause mortality risk by alcohol consumption according to cohort. The Tromsø Study 1994–2020. HR; hazard ratios, are based on cox proportional hazard models with repeated measures of alcohol consumption. All time-varying scores were updated in 2001, 2007–08, and 2015–16 for those who participated. All estimates are adjusted for education, age and including all listed covariates. End of follow-up on November 25, 2020. Exponentiated coefficients; 95% confidence intervals in brackets. *p < 0.05, **p < 0.01, ***p < 0.001. aIn 1994–95, the seven-item CONOR Mental Health Index (CONOR-MHI) was used, whereas in the three subsequent surveys, the ten-item Hopkins Symptom Check List-10 (HSCL-10) was used bHII measures somatic diseases according to the impact that each condition has on SRH. cSubjects reporting the use of either or both sleeping pills/tranquilisers. In 1994–95, the time frame asked was “during the last two weeks”, while in the three subsequent surveys it was “during the last four weeks”. cSubjects reporting the use of either or both sleeping pills/tranquilisers. In 1994–95, the time frame asked was “during the last 2 weeks”, while in the three subsequent surveys it was “during the last 4 weeks”. [file 13690_2023_1035_MOESM2_ESM.rtf]

S.Table 2 All-cause mortality risk by alcohol consumption according to cohort in the Tromsø4-7
	Pre-War II generation (born before 1946)	Baby Boomers (born after 1946)	
	Women	Men	Women	Men	
Alcohol consumption					
Abstainer, not consumed alcohol last 12 months  	1.29***
[1.16, 1.44]	1.16**
[1.04, 1.30]	2.37*
[1.10, 5.13]	1.84
[0.82, 4.16]	
> 0 < 100 grams ethanol per week 	1	1	1	1	
	(ref.)	(ref.)	(ref.)	(ref.)	
≥ 100 grams ethanol per week  	0.96	0.93	1.17	0.84	
	[0.73, 1.26]	[0.80, 1.08]	[0.51, 2.69]	[0.45, 1.54]	
Self-rated health status					
Poor	1	1	1	1	
	(ref.)	(ref.)	(ref.)	(ref.)	
Fair	0.88	0.71***	0.27**	0.53	
	[0.74, 1.05]	[0.61, 0.84]	[0.10, 0.69]	[0.26, 1.10]	
Good	0.62***	0.55***	0.23**	0.42*	
	[0.51, 0.76]	[0.46, 0.65]	[0.09, 0.60]	[0.19, 0.92]	
Excellent	0.40***	0.40***	0.11**	0.00	
	[0.28, 0.57]	[0.30, 0.53]	[0.03, 0.50]	[0.00]	
Live with a spouse or a partner	0.82***	0.81***	0.76	0.76	
	[0.74, 0.90]	[0.74, 0.90]	[0.42, 1.37]	[0.44, 1.31]	
Mental distressa					
No symptoms  	1	1	1	1	
	(ref.)	(ref.)	(ref.)	(ref.)	
Some symptoms  	1.17*	1.25***	0.93	1.90*	
	[1.02, 1.35]	[1.13, 1.39]	[0.47, 1.82]	[1.02, 3.53]	
Sub-threshold symptoms  	1.09	1.20**	0.53	2.34*	
	[0.93, 1.28]	[1.05, 1.38]	[0.23, 1.23]	[1.16, 4.74]	
Significant symptoms  	1.10	1.30*	0.13*	2.94*	
	[0.90, 1.33]	[1.06, 1.59]	[0.03, 0.65]	[1.14,7.58]	
Physical illness (HII)b	1.04***	1.06***	1.06	1.08	
	[1.03, 1.06]	[1.05, 1.08]	[0.92, 1.23]	[0.94, 1.24]	
Smoking					
Never smoked  	1	1	1	1	
	(ref.)	(ref.)	(ref.)	(ref.)	
>1-20 years  	1.12	1.22*	1.61	0.59	
	[0.95, 1.33]	[1.03, 1.44]	[0.57, 4.50]	[0.19, 1.84]	
>20 years  	1.64***	1.90***	3.40**	2.50**	
	[1.48, 1.83]	[1.67, 2.17]	[1.52,7.58]	[1.31, 4.77]	
Have used pillsc last 2/4 weeks  	0.78***	0.90	1.33	1.08	
	[0.70, 0.86]	[0.80, 1.01]	[0.66, 2.69]	[0.56, 2.09]	
High blood pressure (>140/90mmHg)	1.13*	1.24***	1.07	0.96	
	[1.02, 1.25]	[1.13, 1.35]	[0.59, 1.94]	[0.59, 1.57]	
Body Mass Index					
Lean (<25 kg/m2)	1	1	1	1	
	(ref.)	(ref.)	(ref.)	(ref.)	
Overweight (25-30 kg/m2)  	0.66***	0.72***	0.54	1.60	
	[0.59, 0.74]	[0.66, 0.79]	[0.28, 1.04]	[0.83, 3.11]	
Obese (≥30 kg/m2)	0.68***	0.64***	0.68	1.71	
	[0.60, 0.77]	[0.56, 0.73]	[0.33, 1.41]	[0.83, 3.53]	
Average physical activity per week					
Inactive  	1	1	1	1	
	(ref.)	(ref.)	(ref.)	(ref.)	
<1 Hour  	0.70***	0.70***	1.33	1.07	
	[0.60, 0.82]	[0.61, 0.81]	[0.43, 4.15]	[0.45, 2.57]	
1-2 hours  	0.73***	0.68***	1.04	0.87	
	[0.63, 0.83]	[0.60, 0.77]	[0.34, 3.20]	[0.34, 2.18]	
≥3 hours  	0.81**	0.72***	0.98	1.44	
	[0.71, 0.93]	[0.64, 0.82]	[0.31, 3.11]	[0.59, 3.51]	
N	8343	8278	2251	2236	
HR; hazard ratios, are based on cox proportional hazard models with repeated measures of alcohol consumption. All time-varying scores were updated in 2001, 2007-08, and 2015-16 for those who participated. All estimates are adjusted for education, age and including all listed covariates. End of follow-up on November 25, 2020. 
Exponentiated coefficients; 95% confidence intervals in brackets. * p < 0.05, ** p < 0.01, *** p < 0.001 
aIn 1994-95, the seven-item CONOR Mental Health Index (CONOR-MHI) was used, whereas in the three subsequent surveys, the ten-item Hopkins Symptom Check List-10 (HSCL-10) was used bHII measures physical illness according to the impact that each condition has on SRH. cSubjects reporting the use of either or both sleeping pills/tranquilisers. In 1994-95, the time frame asked was “during the last two weeks”, while in the three subsequent surveys it was “during the last four weeks”. cSubjects reporting the use of either or both sleeping pills/tranquilisers. In 1994-95, the time frame asked was “during the last two weeks”, while in the three subsequent surveys it was “during the last four weeks”.
